# Assessment of self-injection experience in patients with rheumatoid arthritis: psychometric validation of the Self-Injection Assessment Questionnaire (SIAQ)

**DOI:** 10.1186/1477-7525-9-2

**Published:** 2011-01-13

**Authors:** Dorothy Keininger, Geoffroy Coteur

**Affiliations:** 1 UCB Pharma SA, Brussels, Belgium

## Abstract

**Background:**

Subcutaneous self-injection of medication has benefits for the patient and healthcare system, but there are barriers such as dexterity problems and injection anxiety that can prevent self-injection being used effectively. An accurate method of evaluating patients' experiences with self-injection would enable assessment of their success in giving self-injections and the likelihood of them adhering to a self-injection regimen. The aim of this study was to develop a questionnaire to measure overall patient experience with subcutaneous self-injection (the Self-Injection Assessment Questionnaire [SIAQ]), and to investigate its psychometric properties.

**Methods:**

The construct validity and reliability of the SIAQ were tested in patients with rheumatoid arthritis who volunteered to inject certolizumab pegol using a standard syringe during an open-label multinational extension trial of the long-term safety and efficacy of this drug. The SIAQ PRE module was self-completed before the first self-injection, and the POST module was self-completed following each of three fortnightly self-injections.

**Results:**

Ninety-seven patients completed the SIAQ. All items correlated well with their respective domains in confirmatory factor analysis. As predicted, compared with other participants, patients with very low scores (less than 3 out of 10) in PRE causal domains (Feelings about injections and Self-confidence) were significantly less satisfied with their first self-injection, as were patients with a very low score in any POST causal domain (Self-confidence, Feelings about injections, Injection-site reactions and Ease of use), demonstrating known-groups validity. Causal domain scores generally correlated most strongly with the Satisfaction with self-injection domain, supporting convergent validity. The SIAQ demonstrated internal consistency and reproducibility; Cronbach's α and the test-retest coefficient were > 0.70 for all domains. Sensitivity and responsiveness were also shown, where measurable. Each language version showed structural validity.

**Conclusion:**

The SIAQ was demonstrated to be a valid, reliable tool in patients with rheumatoid arthritis.

## Background

Rheumatoid arthritis (RA) is a chronic autoimmune disease that is associated with increased morbidity and mortality, and requires long-term treatment [1,2]. The disease leads to pain, fatigue and impairment in physical function, which limit activities and result in a significant decline in health-related quality of life (HRQoL) [3]. Disease-modifying anti-rheumatic drugs (DMARDs) have been demonstrated to improve physical function and HRQoL [4]. Anti-tumour necrosis factor drugs (anti-TNFs) are biologic DMARDs that are effective in inhibiting disease progression [5,6].

Anti-TNFs can be administered by intravenous infusion or by subcutaneous injection. While intravenous infusion requires commitment to regular clinic visits, subcutaneous injection offers the option of self-administration and is likely to provide a better treatment experience for patients. Patients with chronic diseases who are able to self-inject their medication gain control of their treatment schedule (within the limits imposed by the product label) and their treatment setting, thus allowing greater independence and freedom in their social, domestic and professional lives [7,8]. Self-injection may also offer psychological benefits over administration by healthcare professionals, including improved self-esteem [9,10]. This improved treatment experience may lead to better adherence, improved therapeutic outcomes and improved HRQoL. Removing the need to attend a clinic or hospital for regular injections also brings economic benefits to both the patient and the healthcare system.

There are, however, several potential barriers to self-injection [7,8]. Dexterity problems, commonly found in patients with RA, are potential physical barriers to self-injection. Among patient groups that self-inject, psychological and social barriers, such as injection anxiety, lack of confidence in giving a self-injection, and potential embarrassment associated with self-injecting in public, can also be problematic. One in five people are estimated to experience injection anxiety [11], but this prevalence may be substantially greater in some patient groups such as younger patients and those with a history of fainting following injection [11,12].

There is a need to assess the patients' experience with self-injection, and to gauge their success in giving self-injections and the likelihood of them adhering to a self-injection regimen. The Self-Injection Assessment Questionnaire (SIAQ) (^© ^UCB, Braine L'Alleud, Belgium 2006) was developed to assess overall patient experience with subcutaneous self-injection. It assesses the perceived advantages of self-injection and the potential barriers to self-injection, including psychological barriers, social barriers and physical barriers, as well as satisfaction with self-injection and willingness to continue the treatment by self-injection. The SIAQ was developed using a four-step process involving patients with either RA or Crohn's disease (CD). It was designed to evaluate the patient's perceptions before and after they self-inject, and to be suitable for use in clinical studies. The SIAQ can also be used to guide decisions regarding mode of administration when prescribing an anti-TNF.

The aim of this study was to assess the psychometric properties of the SIAQ and to refine it, if necessary. Data for this evaluation were obtained from patients with active RA who were able, in the opinion of their physicians, to self-inject certolizumab pegol, the only PEGylated anti-TNF treatment, as part of a long-term extension of the Rheumatoid Arthritis PreventIon of structural Damage 2 (RAPID 2) study [13].

## Methods

### Questionnaire development

The initial version of the SIAQ (SIAQ_v1.0_) was developed in accordance with a rigorous process based on the draft Food and Drug Administration (FDA) Guidance on Patient Reported Outcomes [14,15].

**Figure 1:**
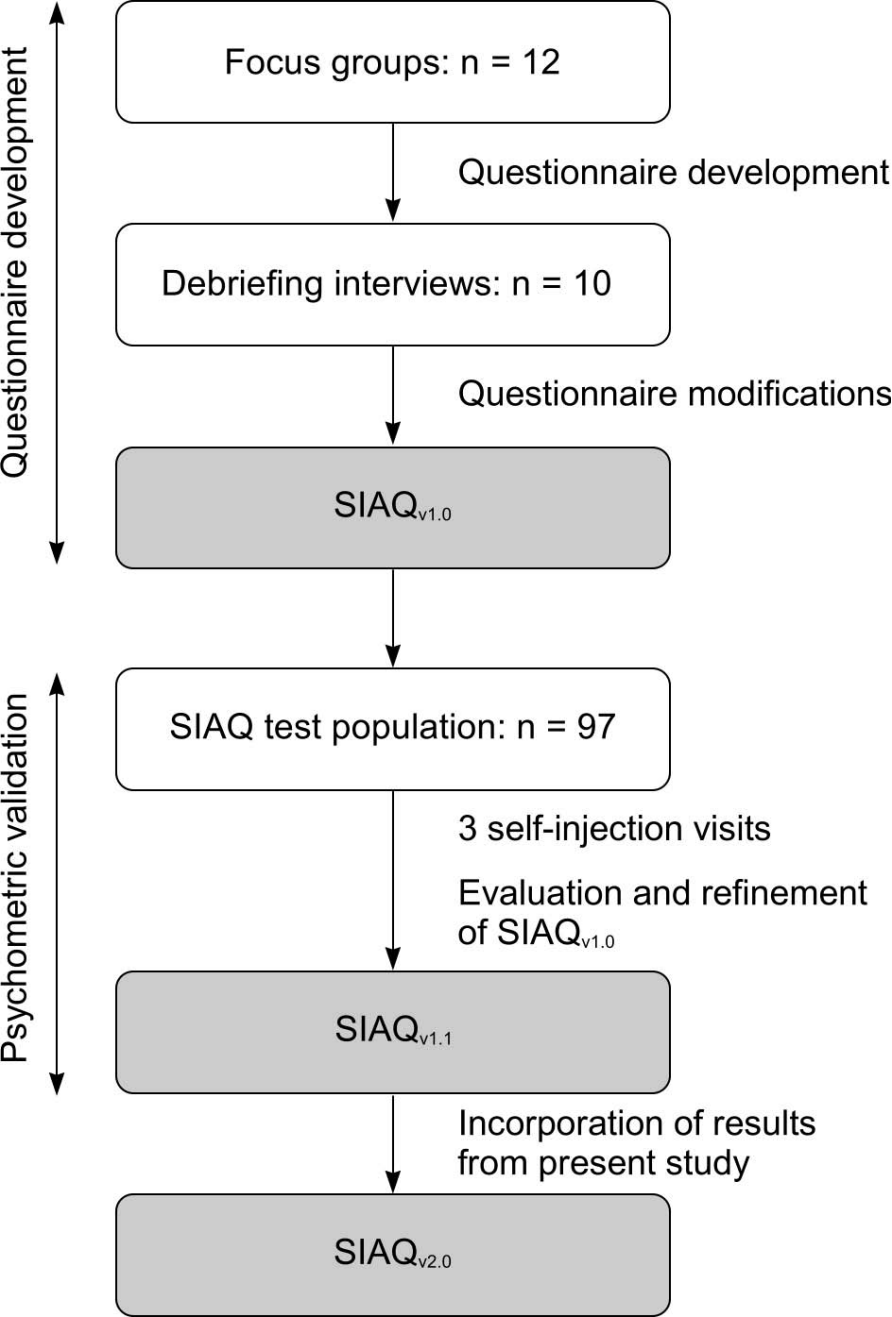
**Study design**.

In-depth structured focus groups and individual interviews were carried out with 12 patients with RA or CD in the USA to understand their perceptions of self-injection (Figure 1). Patients' verbatim comments were also collected to help develop the questionnaire. Six of the patients involved in this stage had RA and six had CD. Ten patients were female, and the mean age was 53 years (range: 24-67 years). The majority of the patients were of North American/European/Caucasian origin (n = 8). Six patients had a college or university degree, seven were in full- or part-time work and six were married.

A qualitative analysis of the in-depth discussions was performed to develop a conceptual model that grouped the concepts that were relevant to the patients. This model was used to construct the questionnaire, with each 'concept' subsequently represented by a hypothetical domain. Items were generated in US English using patients' verbatim comments and grouped within the relevant domains. The SIAQ_v1.0 _was designed as two modules: one that was designed to be completed by patients before their first self-injection (PRE module); and one that was designed to be completed by patients after self-injection (POST module). All items in the PRE module were repeated in the POST module, allowing comparison of patients' feelings about injections before and after self-injection.

**Figure 2:**
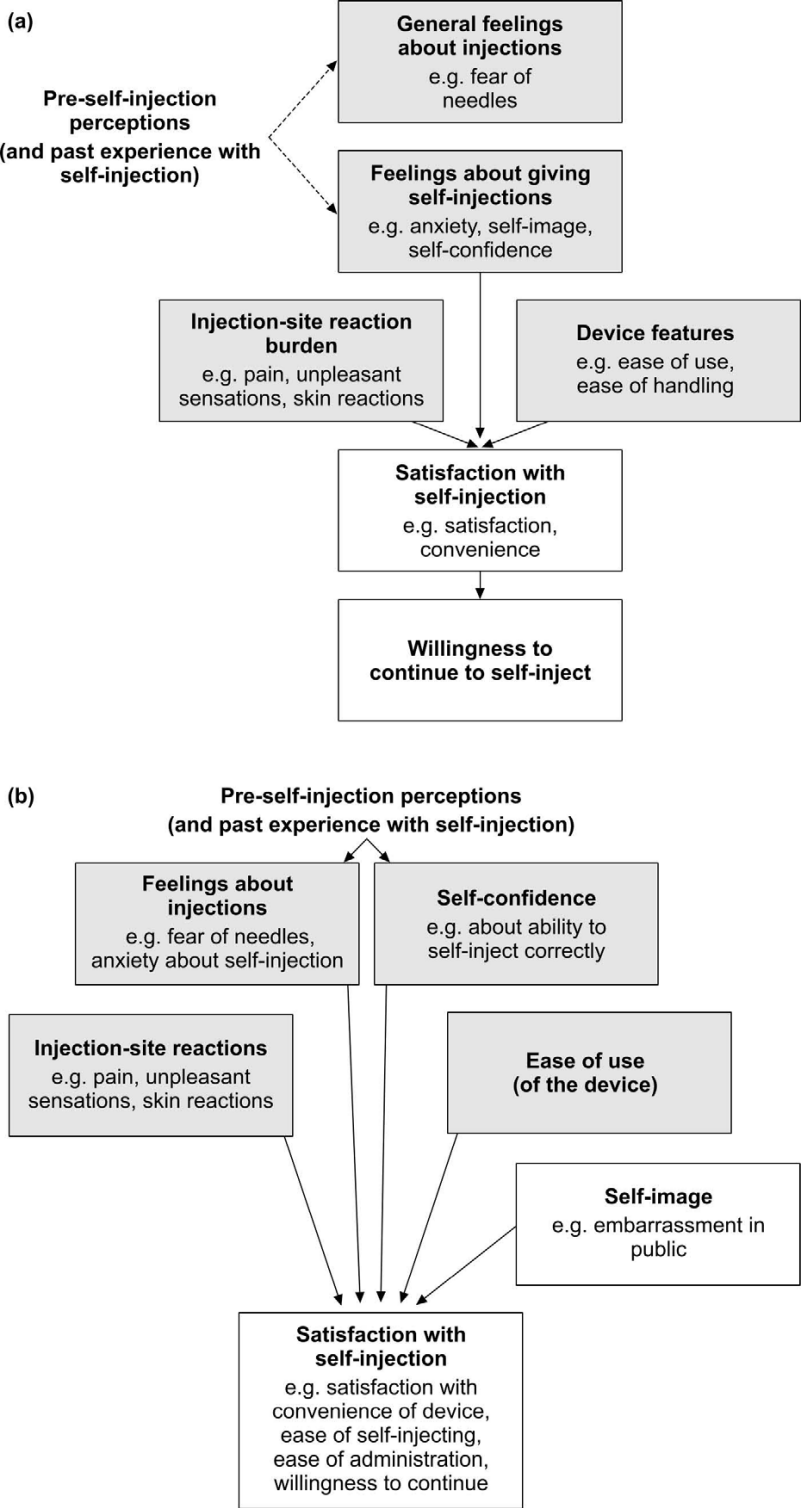
**The hypothetical (a) and refined (b) conceptual models of the Self-Injection Assessment Questionnaire^© ^version 1.0 (SIAQ_v1.0_)**. Causal concepts are indicated in light grey boxes

Eight items were generated for the PRE module, which were grouped into three hypothetical domains (General feelings about injections, Feelings about giving self-injections, and Satisfaction with self-injection) (Table 1). Twenty-three items were generated for the POST module, with two of these being divided into eight sub-items (Table 2). The items were grouped into six hypothetical domains (General feelings about injections, Feelings about giving self-injections, Injection-site reaction burden, Device features, Satisfaction with self-injection, and Willingness to continue to self-inject). The first four domains of the POST module were hypothesized to be causal concepts according to the conceptual model, because they were considered determining factors of satisfaction with self-injection and willingness to continue (Figure 2a).

**Table 1:** The structure of the PRE module of the Self-Injection Assessment Questionnaire^© ^(SIAQ)_v1.0 _and the SIAQv1.1.

Items	Hypothetical domains (v1.0)	Refined domains (v1.1)
1. In general, how afraid are you of needles?	General feelings about injections	Feelings about injections
		
2. In general, how afraid are you of having an injection?		
	
3. How anxious do you feel about giving yourself an injection?	Feelings about giving self-injections	
		
4. How confident are you about giving yourself an injection in the right way?		Self-confidence
		
5. How confident are you about giving yourself an injection in a clean and sterile way?		
		
6. How confident are you about giving yourself an injection safely?		
		
7. Does your current way of taking your medication make you feel in control of your disease?		**DELETED**

8. Overall, how satisfied are you with your current way of taking your medication?	Satisfaction with self-injection	Satisfaction with self-injection

**Table 2:** The structure of the POST module of the Self-Injection Assessment Questionnaire^© ^(SIAQ)_v1.0 _and the SIAQv1.1.

Items and sub-items	Hypothetical domains (v1.0)	Refined domains (v1.1)
1. In general, how afraid are you of needles?	General feelings about injections	Feelings about injections
		
2. In general, how afraid are you of having an injection?		
	
3. How anxious do you feel about giving yourself an injection?	Feelings about giving self-injections	
		
4. How embarrassed would you feel if someone saw you with the self-injection device?		Self-image
		
5. How confident are you about giving yourself an injection in the right way?		Self-confidence
		
6. How confident are you about giving yourself an injection in a clean and sterile way?		
		
7. How confident are you about giving yourself an injection safely?		
		
8. Does your current way of taking your medication (self-injection) make you feel in control of your disease?		**DELETED**

9a. During and/or after the injection, how bothered were you by pain?	Injection-site reaction burden	Injection-site reactions
		
9b. During and/or after the injection, how bothered were you by burning sensation?		
		
9c. During and/or after the injection, how bothered were you by cold sensation?		
		
10a. During and/or after the injection, how bothered were you by itching at the injection site?		
		
10b. During and/or after the injection, how bothered were you by redness at the injection site?		
		
10c. During and/or after the injection, how bothered were you by swelling at the injection site?		
		
10d. During and/or after the injection, how bothered were you by bruising at the injection site?		
		
10e. During and/or after the injection, how bothered were you by hardening at the injection site?		

11. How much do you agree or disagree with the following: the cap is easy to remove.	Device features	Ease of use
		
12. How much do you agree or disagree with the following: the device fits comfortably in my hand.		
		
13. How much do you agree or disagree with the following: I can easily depress the plunger or button on the device.		
		
14. How much do you agree or disagree with the following: I can administer the injection without any help.		
		
15. How much do you agree or disagree with the following: the self-injection device is easy to use.		

16. How easy was it to give yourself an injection?	Satisfaction with self-injection	Satisfaction with self-injection
		
17. How satisfied are you with how often you give yourself an injection?		
		
18. How satisfied are you with the time it takes to inject the medication?		
		
19. Overall, how satisfied are you with your current way of taking your medication (self-injection)?		
		
20. Overall, how convenient is the self-injection device?		
		
21. Overall, how comfortable is the injection?		**DELETED**

22. After this study, would you choose to continue self-injecting your medication?	Willingness to continue to self-inject	Satisfaction with self-injection
		
23. After this study, how confident would you be to give yourself injections at home?		

Content validity and acceptability of the items were evaluated through cognitive debriefing interviews that were conducted with a new group of patients. Five patients with CD and five patients with RA took part in these cognitive debriefing interviews. Six of these participants were female, and the mean age was 43 years (range: 20-63 years).

The original US English language version of the SIAQ_v1.0 _was translated into Czech and Polish. Linguistic adaptation was undertaken by translation professionals using a validated procedure that involved five steps [16]. These were: forward translation by two independent translators; reconciliation of the two translations; backward translation by an independent translator; comparison of the source questionnaire with the backward translation by a local consultant and the translation professionals; and cognitive debriefing with five patients with either CD or RA. Minor modifications were made following this validation procedure to improve the translatability of the questionnaire.

### Psychometric validation

#### Study design

The psychometric properties of the SIAQ were investigated in adult patients in the USA, Poland and the Czech Republic who were enrolled in a multicentre, open-label, single-arm extension of the RAPID 2 trial assessing the long-term safety and efficacy of certolizumab pegol in addition to methotrexate in the treatment of active RA [13]. All participating patients provided written informed consent. Patients were not given any incentives to complete the study.

Participants were invited to self-inject 400 mg certolizumab pegol as two 1-mL injections at separate injection sites (lateral abdominal wall and upper outer thigh were suitable sites) using a pre-filled standard syringe. Patients who agreed to participate and who were judged by the investigators to be capable of safely and effectively administering the treatment were included in the self-injection study. Patient capability was not pre-defined and was left to the treating physician's discretion. The self-injections were carried out at three consecutive visits planned every 2 weeks. Training was provided at each visit, along with written instructions on the recommended subcutaneous injection technique. Patients self-injected under supervision of the site staff to ensure that the study medication was properly and safely injected. Patients rated their perceptions about self-injections using the SIAQ_v1.0 _before the first self-injection and at each self-injection visit (SIV). All patients who completed at least one of the three SIVs and one POST module assessment were included in the analysis.

Participation was voluntary and patients could withdraw from the self-injection study at any time and continue with the original study. The study protocol was approved by the appropriate Ethics Committees and Institutional Review Boards.

#### Questionnaire administration and scoring

The two modules of the SIAQ_v1.0 _were completed by patients while alone in a quiet environment. The PRE module was completed immediately before the first self-injection and the POST module was completed 20-40 minutes after dosing at each SIV, based on the combined experience of the two injections at that visit. There is no stipulated recall period for the SIAQ, because the recall period varies from one domain to another. For example, items from the Injection-site reaction burden domain refer to the patient's experience during or after the injection, whereas items from the General feelings about injections domain refer to general attitudes.

Patients rated each item of the SIAQ on a 5-point semantic Likert-type scale, where a score of 1 corresponded to the patient's worst experience and a score of 5 corresponded to the patient's best experience. Item scores were transformed to obtain a score ranging from 0 (worst experience) to 10 (best experience) for each item. The domain score was the mean of the item scores included in the domain. Domain scores were calculated only if at least half of the domain items were completed.

#### Initial evaluation and refinement of the SIAQ_v1.0_

Patient data obtained with the POST module at the first SIV (SIV-1) were used for an exploratory factor analysis to evaluate the hypothetical structure of the SIAQ_v1.0_. Appropriate modifications to the SIAQ structure and conceptual model were subsequently made to produce the SIAQ version 1.1 (SIAQ_v1.1_). The varimax method of factor rotation was used and factors with an eigenvalue > 1 were retained. The psychometric properties of the SIAQ_v1.1 _(construct validity and reliability [17]) were investigated using data obtained from the relevant items of the SIAQ_v1.0_, but applied to the new conceptual model and questionnaire structure.

#### Construct validity of the SIAQ_v1.1_

To assess the extent to which the questionnaire measured the intended concepts, the construct validity of the refined questionnaire (SIAQ_v1.1_) was examined using confirmatory factor analysis, goodness-of-fit indices, known-groups validity and convergent validity.

Two confirmatory factor analyses were performed to evaluate the construct validity of both the PRE and the POST SIAQ_v1.1 _modules, using item scores from the PRE module and from SIV-3, respectively. Items that correlated with their respective domain with a coefficient of ≥ 0.5 were considered to have good construct validity [18].

The goodness-of-fit of the data to the new structure was assessed using four fit indices: the Bentler's comparative fit index (CFI), which ranges from 0 to 1, with higher values indicating better fit and where 0.90 is considered an adequate fit [19]; the normed fit index (NFI) where a value of 0.90-0.95 is acceptable and a value of > 0.95 is good [19];

the root mean square error of approximation (RMSEA), where a value of < 0.05 indicates a good fit, 0.05-0.1 a reasonable fit and > 0.1 a poor fit [20]; and the root mean square residual (RMR), where a value of < 0.05 indicates a good fit [21].

Known-groups validity is demonstrated when an instrument is sensitive enough to discriminate between groups of individuals known to be different according to the variables being measured [22]. From the refined conceptual model (Figure 2b), it was hypothesized that patients with greater fear or anxiety before the first self-injection (PRE Feelings about injections domain scores ≤ 2), lower self-confidence before the first self-injection (PRE Self-confidence domain scores ≤ 2) or a worse experience in any of the causal domains after the first self-injection (POST SIV-1 domain scores ≤ 2) would have a lower level of satisfaction with their first self-injection than patients with higher domain scores (> 2). Student *t*-tests for independent samples were performed to assess potential differences in Satisfaction with self-injection scores at SIV-1.

Convergent validity is demonstrated when two or more measures that are theoretically related are actually observed to be related [22]. Convergent validity was investigated, using data from SIV-1, by evaluating whether causal domains (Feelings about injections, Self-confidence, Injection-site reactions, Ease of use, and Self-image) were more strongly correlated with the Satisfaction with self-injection domain than with each other, as hypothesized by the refined conceptual model. The correlation between the Satisfaction with self-injection domain score and the combined mean of all causal domains was also evaluated, and was expected to be high (> 0.5).

#### Reliability of the SIAQ_v1.1_

Reliability was assessed to ascertain the extent to which the questionnaire yielded the same score when expected. This reliability assessment comprised measurement precision (floor and ceiling effects, and internal consistency), reproducibility (test-retest reliability), sensitivity (between-groups discrimination), and responsiveness (within-groups discrimination). The presence of floor (an excess of minimum scores) and ceiling (an excess of maximum scores) effects were examined by calculating the distribution of domain scores at SIV-1. Floor or ceiling effects indicate that a domain might have poor sensitivity or responsiveness, as further deterioration or improvement will not be measurable in patients with low or high scores, respectively [23].

Internal consistency was evaluated using Cronbach's α coefficient [24] to determine the extent to which item scores correlated with dimension scores before and after the first self-injection. An α coefficient above 0.70 suggests good internal consistency and reliability [25].

Test-retest reliability is a measure of the stability of the instrument under different conditions with the same rater. The intraclass correlation coefficient (ICC) was used to measure the strength of agreement between domain scores as assessed at SIV-2 and SIV-3. Overall perceptions about self-injections were expected to change earlier in the study and be maintained later in the study, so the SIV-2 and SIV-3 were expected to remain fairly stable. A test-retest coefficient above 0.70 was considered acceptable [26].

The sensitivity of an instrument is its ability to detect differences between patients or groups of patients. The single-arm design of the study limited the assessment of sensitivity, which could be tested only for the Satisfaction with self-injection domain, as per the known-groups construct validity analysis (see above).

Responsiveness relates to the ability of an instrument to detect changes when a patient improves or deteriorates [22]. To test responsiveness, the two domain scores that were expected to improve (Feelings about injections and Self-confidence) were compared using *t*-tests for paired samples before the first self-injection and after SIV-3. The effect size was also calculated to assess the magnitude of these expected improvements [27].

#### Language and country effects

SIAQ_v1.1 _domain scores were compared among language versions at SIV-1 using analysis of covariance (ANCOVA) with country and sex as factors, and age and baseline physical functioning as covariates. Baseline physical functioning was assessed using the Health Assessment Questionnaire disability index [28].

A convergent-discriminant analysis was performed on the Czech, Polish and US English versions of the SIAQ to investigate the strength of correlation between item scores and domain scores at SIV-3. Items would be expected to be more strongly correlated with items in the same domain than with items from different domains if the conceptual model was supported in these language versions.

## Results

### Study population

Of the 98 patients who entered the study, 97 performed at least one self-injection and were therefore included in the analysis (10 from the USA, 30 from the Czech Republic and 57 from Poland). One patient was unable to administer a self-injection. Demographic characteristics of the patients are shown in Table 3. These characteristics were representative of the RAPID 2 study population.

**Table 3:** Demographic characteristics of the SIAQ^© ^patient population.

	SIAQ patient population N = 97
**Age**	
Mean	51.8 years (SD: 9.7)
Range	28-69 years
**Sex**	
Female	76.3% (n = 74)
**Body mass index**	
Mean	27.5 kg/m^2 ^(SD: 5.1)
**Ethnicity**	
Caucasian	99.0% (n = 96)

SD, standard deviation; SIAQ, self-injection assessment questionnaire.

### SIAQ completion rates

All 97 patients completed the PRE module, and each item was completed by at least 99.0% of the analysis population. The number of patients present at each SIV was 97 (100.0%), 96 (99.0%) and 92 (94.8%) at SIV-1, SIV-2 and SIV-3, respectively, and each patient filled in at least 95% (95.8-100.0%) of POST module items at each visit. Ninety-one patients (93.8%) completed all three self-injections. Of the remaining six patients, four missed one entire visit (no study drug was administered), one patient did not perform the third self-injection because of feeling "too tired", and the last patient did not perform the third self-injection because the injection was "too painful on thumb".

### Initial evaluation and refinement of SIAQ_v1.0_

Exploratory factor analysis using data from SIV-1 confirmed most of the relationships between items and domains that were hypothesized in the original conceptual model (Additional file 1, Figure 2a). Five factors were extracted that explained 86.8% of the total variance in the sample.

Several modifications were made to the conceptual model and questionnaire structure based on the analysis of the correlation matrix (POST module) together with the cognitive debriefing transcripts (Table 2; Figure 2). The corresponding modifications to the PRE module were also made (Table 1).

Items relating to how 'comfortable' the injection was (item 21) and to notions of 'feeling in control' (item 8) were not clearly or universally understood by patients. The data for these items correlated to a similar degree with more than one factor, indicating that they did not relate to any single domain in particular. For some patients, the term 'comfortable' related to the injection itself, whereas for others it related to the experience of self-injecting. Similarly, different patients related 'feeling in control' to various concepts, such as efficacy of the medication, comfort, confidence or satisfaction. These items were therefore deleted.

The General feelings about injections domain (items 1 and 2) was expanded to include item 3 from the hypothetical Feelings about giving self-injections domain and was renamed Feelings about injections in the refined version. This was done because the three items in this new domain were conceptually related and showed high correlation as one specific factor. Items 5-7 in the original Feelings about giving self-injections domain were renamed the Self-confidence domain because they were restricted to items related to self-confidence with self-injection. Item 4 (embarrassment about being seen with the injection device) correlated with more than one factor, but was understood clearly by patients. This item was therefore retained, but was removed from the Feelings about giving self-injections domain to become a single-item domain, Self-image.

Exploratory factor analysis supported the hypothetical domains Injection-site reaction burden and Device features, with the exception of item 14 in the latter domain (concerning ability to administer the injection without help), which correlated with more than one factor. However, this item was retained in the refined version because it was clearly understood by patients during cognitive debriefing. The two domains were renamed Injection-site reactions and Ease of use in the refined version.

Finally, items from the hypothetical Willingness to continue to self-inject domain (items 22 and 23) were re-grouped within the Satisfaction with self-injection domain, because these items correlated highly with the factor that represented this domain. The Willingness to continue to self-inject concept was therefore removed from the refined version.

The refined SIAQ (SIAQ_v1.1_), consisted of a PRE module with seven items grouped into three domains (Feelings about injections, Self-confidence and Satisfaction with self-injection), and a POST module with 21 items (two of which were divided into eight sub-items), grouped in four principal causal domains (Feelings about injections, Self-confidence, Injection-site reactions, Ease of use) plus a single item assessing Self-image. These domains are determining factors of the sixth concept, assessed by the Satisfaction with self-injection domain (Figure 2b).

### Construct validity of SIAQ_v1.1_

Confirmatory factor analyses of the SIAQ_v1.1 _using item scores taken before the first self-injection (PRE module) and at SIV-3 (POST module) showed that all items correlated with their respective domains with coefficients ≥ 0.5, indicating good construct validity (POST module, 0.5 to > 0.99; PRE module, 0.64 to > 0.99). These two analyses confirmed the structure of the POST module that was suggested by the exploratory factor analysis, and confirmed that the modifications made to the POST module also applied to the PRE module. The goodness-of-fit of the data to the questionnaire structure was reasonable given the limited data. Bentler's CFI was 0.99 for the PRE module and 0.88 for the POST module; the NFI was 0.96 for the PRE module and 0.76 for the POST module; the RMSEA was 0.06 for the PRE module and 0.09 for the POST module; and the RMR was 0.04 for the PRE module and 0.08 for the POST module.

Known-groups validity was demonstrated. As expected, patients with higher fear or lower confidence levels before their first self-injection experienced lower satisfaction or a worse experience in any of the causal domains after their first injection than did patients who were less afraid or more confident about self-injecting before SIV-1 (Table 4).

**Table 4:** Satisfaction with self-injection grouped by Self-Injection Assessment Questionnaire^© ^version 1.1 (SIAQ_v1.1_) causal domain score

Domain-score group	n	Satisfaction score at first self-injection visit	
		Mean (SD)	*p*
**Overall**			
Causal domain scores all > 2 at first injection (best scores)	65	7.09 (1.50)	-
Any causal domain score ≤ 2 at first self-injection (worst scores)	32	6.14 (1.69)	0.009
**Confidence**			
More confident *before *first injection (domain score > 2)	47	7.47 (1.37)	-
Less confident *before *first injection (domain score ≤ 2)	36	6.09 (1.60)	< 0.001
**Anxiety**			
Less afraid/anxious *before *first injection (domain score > 2)	63	7.32 (1.51)	-
More afraid/anxious *before *first injection (domain score ≤ 2)	14	5.47 (1.21)	< 0.001

SD, standard deviation.

As hypothesized, causal domains generally correlated with the Satisfaction with self-injection domain (Table 5), thus supporting convergent validity. Except for Injection-site reactions, all causal domain scores were more strongly correlated with the Satisfaction with self-injection domain score than with other domain scores at SIV-1. The correlation between the Satisfaction with self-injection domain score and the combined mean of all causal domain scores was strong (0.67), and Satisfaction with self-injection was more strongly correlated with this mean than with any single causal domain score.

**Table 5:** Correlation between Self-Injection Assessment Questionnaire^© ^version 1.1 (SIAQ_v1.1_) domain scores at first self-injection visit

Domains	Feelings about injections	Self-confidence	Injection-site reactions	Ease of use	Satisfaction with self-injection
Feelings about injections	-	0.21	0.42	0.40	0.62
Self-confidence	0.21	-	0.12	0.32	0.33
Injection-site reactions	0.42	0.12	-	0.38	0.39
Ease of use	0.40	0.32	0.38	-	0.52
Satisfaction with self-injection	0.62	0.33	0.39	0.52	-
Mean of causal domains	-	-	-	-	0.67

### Reliability of the SIAQ_v1.1_

The reliability of the SIAQ_v1.1 _was demonstrated in this study. Cronbach's α coefficient was above 0.70 for all PRE and POST module domains, demonstrating good internal consistency (Table 6). The test-retest reliability analysis indicated good stability and reproducibility between SIV-2 and SIV-3, with all domain ICCs being above 0.70 (Table 6).

**Table 6:** Self-Injection Assessment Questionnaire^© ^version 1.1 (SIAQ_v1.1_) reliability evaluations

Domains	Proportion at floor (%)	Proportion at ceiling (%)	Cronbach's α coefficient	Intraclass correlation coefficient
**PRE module**				
Feelings about injections	0.0	17.5	0.89	-
Self-confidence	3.1	1.0	0.82	-
Satisfaction with self-injection	2.1	14.4	-	-
**POST module**				
Feelings about injections	0.0	27.8	0.92	0.93
Self-confidence	6.3	6.3	0.90	0.82
Injection-site reactions	0.0	24.0	0.89	0.86
Ease of use	0.0	21.6	0.85	0.79
Satisfaction with self-injection	0.0	4.1	0.90	0.89

Responsiveness was evaluated by comparing mean scores before SIV-1 (PRE module) and after SIV-3 (POST module) in the Feelings about injections and Self-confidence domains. A significant improvement in mean domain scores was observed (Feelings about injections: 6.99 [SIV-1] vs 7.53 [SIV-3], *p *= 0.004; Self-confidence: 5.26 [SIV-1] vs 5.82 [SIV-3], *p *= 0.015). Effect sizes were small for both domains (0.23 for Feelings about injections, and 0.26 for Self-confidence), indicating that small differences over time could be detected.

The Satisfaction with self-injection domain demonstrated sensitivity in discriminating between patients with lower and higher scores in the PRE module Self-confidence and Feelings about injections domains or in any of the POST module causal domains at SIV-1 (Table 4). A good distribution of maximum and minimum values was observed for all PRE module domains and for the Self-confidence and Satisfaction with self-injection domains of the POST module, indicating no limitation in the sensitivity of these domains. However, some ceiling effects were seen in three domains of the POST module (Feelings about injections, Injection-site reactions and Ease of use) (Table 6).

### Variations in country/language domain scores

There were significant differences among language versions of the SIAQ in the mean scores of the Injection-site reactions and Satisfaction with self-injection domains at SIV-1. Patients in the Czech Republic had lower Injection-site reactions scores than those in the USA and Poland, although the difference was small (8.46 vs 9.10 in Poland and 9.16 in the USA, *p *= 0.033). Patients from the USA had the highest Satisfaction with self-injection scores (8.75 vs 6.25 in the Czech Republic and 6.70 in Poland, *p *< 0.001). Sex and age had no significant impact on POST module domain scores.

Convergent-discriminant analysis using available data from each language version of the SIAQ_v1.1 _at SIV-3 showed that items generally correlated most strongly with their respective domains. In the Czech version, 26 out of 27 items (with the exception of 'bothered by pain') correlated most strongly with their respective domains. In the Polish version, scores for all 27 items and sub-items correlated most strongly with the appropriate domain score. In the US English version, all but three items correlated most strongly with their respective domains. The exceptions were 'bothered by burning', 'satisfied with how often' and 'choose to continue'.

## Discussion

This study has shown the SIAQ_v1.1 _to be a valid and reliable tool to evaluate patients' feelings about, and experiences with, self-injection. The SIAQ_v1.1 _demonstrated good internal consistency and reproducibility. Preliminary data also indicated good sensitivity and responsiveness for the SIAQ_v1.1 _domains that could be evaluated. Confirmatory factor analysis and goodness-of-fit indices confirmed that the SIAQ_v1.1 _structure fitted the data reasonably. Hypothetical relationships from the conceptual model between causal domains and patient satisfaction with self-injection were supported using known-groups and convergent validities.

The conceptual model of the SIAQ highlighted the impact of factors that can be considered 'causal', such as usability and the burden of injection-site reactions, on patients' overall treatment experience and satisfaction with self-injections. This suggests that devices that are better designed for patients, especially for patients with disabling conditions, would improve the overall experience with the self-injection of treatment and that this might translate into greater adherence.

A significant proportion of patients had the best possible score for the Injection site reactions domain, which suggests that certolizumab pegol was well tolerated by patients. This is in line with the findings of previous studies [13,29,30], and is likely to further encourage adherence to the treatment regimen. Additional studies with other treatments are needed to evaluate whether the Injection site reactions domain score has an intrinsic ceiling effect.

Some ceiling effects were also observed for the Feelings about injections and Ease of use domains. For these domains, patients' experiences were good to excellent: they had few negative feelings about needles or injections and found the pre-filled syringe easy to use. The fact that participation in the study was voluntary may explain the ceiling effects in these domains, as patients who were more afraid of injections or who had severe limitations in dexterity probably did not opt to participate.

The key strength of the SIAQ is that, unlike other self-injection questionnaires [31-35], it is not disease- or treatment-specific. Once the final version has been fully validated, it will be the first questionnaire that can be used to assess self-injection in a range of therapy areas. Another important strength is that the SIAQ was developed and validated in line with best practice, including the FDA guidelines [14,15,17]. The original version of the questionnaire also underwent rigorous linguistic adaptation to resolve any potential translation problems early in the process, before it was administered or further refined. This is an unusual step, but one that was nevertheless helpful and resulted in a few minor modifications. The convergent-discriminant analysis confirmed the structural validity of each language version of the SIAQ. The differences in some domain scores between countries could therefore be due to intrinsic differences in the underlying concepts being evaluated. This highlights the need to consider geographical location when analysing results from multinational studies using the SIAQ.

Potential limitations of the study include the possible selection bias in the patient population. Participants were volunteers who were judged capable of administering subcutaneous self-injections by physicians. In addition, data on previous exposure to self-injection were not collected, so it is possible that not all patients were self-injection naïve. The majority of the volunteers were not fearful or anxious of injections, and had few problems using the pre-filled syringe, which may have produced ceiling effects. Further investigations with a more representative population are needed to assess whether these ceiling effects translate into a decrease in sensitivity.

Another limitation was the small sample size, which may reduce the stability of the factor analysis [36] and limit the interpretation of the goodness-of-fit indices. An increased sample size might also have provided more confidence in determining potential country or language effects. The small sample size and the exploratory nature of the statistical tests means that the *p *values reported in the study should be used to guide interpretation rather than being definitive answers. In addition, the single-arm design and limited efficacy data meant that the evaluation of sensitivity and responsiveness could be applied only to testing logical hypotheses derived from the conceptual model through analysing differences in domain scores. Another potential limitation of the study is that the exploratory and confirmatory factor analyses were performed using the same patient population. This may have led to an overestimation of fit in the confirmatory factor analyses.

Further studies are needed to validate the final version of the questionnaire, SIAQ_v2.0 _(Additional file 2), which incorporates modifications resulting from the findings of this study. These studies should include an assessment of whether extending the response options in the Ease of use domain from 5 to 6 points would enable more subtle feelings of the patient to be captured and ceiling effects to be reduced [22]. Rephrasing items in this domain so that statements are not positive should reduce acquiescence bias [37]. Future linguistic validation will assess the relevance of the Self-image domain in the context of clinical trials. Finally, psychometric validation is required in larger trials with other patient populations to examine the performance of SIAQ_v2.0 _with different self-injection variables (disease, treatment, patient ability and injection device).

## Conclusions

The findings of this study support the validity and reliability of the SIAQ in assessing overall patient experience with subcutaneous self-injection in RA. The factors influencing patients' experience and satisfaction with their self-injected treatment, such as usability and burden from injection-site reactions, were confirmed in the conceptual model of the SIAQ. This suggests that a device with a higher usability or a treatment with fewer injection-site reactions would empower patients who self-inject their medication and provide them with a better overall treatment experience. Further studies will need to be performed to validate the final version of the questionnaire, and before it can be considered to be a trans-disease questionnaire.

## Competing interests

Dorothy Keininger and Geoffroy Coteur are employed by UCB Pharma. The study was funded by UCB Pharma.

## Authors' contributions

DK and GC led the development of the questionnaire and the design, analysis, and interpretation of the psychometric validation. Both authors read and approved the final manuscript.

## Supplementary Material

Additional file 1 **The structure of the Self-Injection Assessment Questionnaire^© ^(SIAQ) before and after exploratory factor analysis**. a) POST module b) PRE module Click here for file

Additional file 2 **Self-Injection Assessment Questionnaire^© ^(SIAQ) version 2.0**. Click here for file
