# Supplementary material for: Assessment of self-injection experience in patients with rheumatoid arthritis: psychometric validation of the Self-Injection Assessment Questionnaire (SIAQ)
Source: Health Qual Life Outcomes. 2011 Jan 13;9:2. doi: 10.1186/1477-7525-9-2 (PMC3027089; doi:10.1186/1477-7525-9-2)
Supplement: Additional file 1 — The structure of the Self-Injection Assessment Questionnaire© (SIAQ) before and after exploratory factor analysis. a) POST module b) PRE module [file 1477-7525-9-2-S1.DOC]

## Additional file 1. The structure of the Self-Injection Assessment Questionnaire© (SIAQ) before and after exploratory factor analysis

## a) POST module

| **Items and sub-items** | **Factor 1** | **Factor 2** | **Factor 3** | **Factor 4** | **Factor 5** | **Hypothetical domains (v1.0)** | **Refined domains (v1.1)** |
| --- | --- | --- | --- | --- | --- | --- | --- |
| 1. In general, how afraid are you of needles? | 0.256 | **0.331** | 0.200 | 0.075 | **0.784** | General feelings about injections | Feelings about injections |
| 2. In general, how afraid are you of having an injection? | 0.266 | **0.303** | 0.098 | 0.060 | **0.826** |  |  |
| 3. How anxious do you feel about giving yourself an injection? | **0.477** | 0.294 | 0.156 | 0.021 | **0.665** | Feelings about giving self-injections |  |
| 4. How embarrassed would you feel if someone saw you with the self-injection device? | **0.423** | **0.321** | 0.184 | 0.024 | **0.327** |  | Self-image |
| 5. How confident are you about giving yourself an injection in the right way? | 0.209 | –0.032 | 0.124 | **0.828** | 0.120 |  | Self-confidence |
| 6. How confident are you about giving yourself an injection in a clean and sterile way? | 0.048 | 0.098 | 0.174 | **0.837** | –0.036 |  |  |
| 7. How confident are you about giving yourself an injection safely? | 0.241 | 0.066 | 0.105 | **0.851** | 0.089 |  |  |
| 8. Does your current way of taking your medication (self-injection) make you feel in control of your disease? | **0.467** | 0.085 | 0.092 | **0.393** | –0.015 |  | **DELETED** |
| 9a. During and/or after the injection, how bothered were you by pain? | **0.309** | **0.518** | 0.063 | 0.132 | 0.228 | Injection-site reaction burden | Injection-site reactions |
| 9b. During and/or after the injection, how bothered were you by burning sensation? | 0.004 | **0.478** | 0.153 | 0.135 | 0.172 |  |  |
| 9c. During and/or after the injection, how bothered were you by cold sensation? | 0.263 | **0.685** | 0.104 | 0.114 | 0.241 |  |  |
| 10a. During and/or after the injection, how bothered were you by itching at the injection site? | 0.266 | **0.683** | –0.074 | 0.163 | 0.137 |  |  |
| 10b. During and/or after the injection, how bothered were you by redness at the injection site? | 0.154 | **0.799** | 0.133 | –0.048 | 0.029 |  |  |
| 10c. During and/or after the injection, how bothered were you by swelling at the injection site? | 0.226 | **0.818** | 0.107 | 0.088 | 0.133 |  |  |
| 10d. During and/or after the injection, how bothered were you by bruising at the injection site? | 0.055 | **0.616** | 0.227 | –0.149 | 0.048 |  |  |
| 10e. During and/or after the injection, how bothered were you by hardening at the injection site? | 0.034 | **0.806** | 0.120 | –0.042 | 0.102 |  |  |
| 11. How much do you agree or disagree with the following: the cap is easy to remove. | –0.003 | 0.154 | **0.689** | 0.075 | 0.040 | Device features | Ease of use |
| 12. How much do you agree or disagree with the following: the device fits comfortably in my hand. | 0.167 | 0.182 | **0.721** | 0.078 | 0.044 |  |  |
| 13. How much do you agree or disagree with the following: I can easily depress the plunger or button on the device. | 0.127 | 0.118 | **0.781** | 0.208 | 0.145 |  |  |
| 14. How much do you agree or disagree with the following: I can administer the injection without any help. | **0.479** | 0.154 | **0.494** | 0.138 | **0.320** |  |  |
| 15. How much do you agree or disagree with the following: the self-injection device is easy to use. | **0.415** | 0.098 | **0.680** | 0.083 | 0.154 |  |  |
| 16. How easy was it to give yourself an injection? | **0.656** | 0.022 | 0.219 | 0.127 | **0.364** | Satisfaction with self-injection | Satisfaction with self-injection |
| 17. How satisfied are you with how often you give yourself an injection? | **0.711** | 0.227 | 0.002 | 0.167 | 0.172 |  |  |
| 18. How satisfied are you with the time it takes to inject the medication? | **0.802** | 0.194 | 0.049 | 0.215 | –0.001 |  |  |
| 19. Overall, how satisfied are you with your current way of taking your medication (self-injection)? | **0.797** | 0.158 | –0.012 | 0.154 | 0.216 |  |  |
| 20. Overall, how convenient is the self-injection device? | **0.674** | 0.249 | **0.329** | 0.156 | 0.019 |  |  |
| 21. Overall, how comfortable is the injection? | **0.474** | **0.351** | 0.281 | 0.155 | 0.164 |  | **DELETED** |
| 22. After this study, would you choose to continue self-injecting your medication? | **0.672** | 0.216 | 0.110 | –0.073 | 0.200 | Willingness to continue to self-inject | Satisfaction with self-injection |
| 23. After this study, how confident would you be to give yourself injections at home? | **0.719** | 0.003 | 0.223 | 0.062 | 0.183 |  |  |

Factor correlations > 0.3 are indicated in bold.

b) PRE module

| **Items** | **Hypothetical domains (v1.0)** | **Refined domains**  **(v1.1)** |
| --- | --- | --- |
| 1. In general, how afraid are you of needles? | General feelings about injections | Feelings about injections |
| 2. In general, how afraid are you of having an injection? |  |  |
| 3. How anxious do you feel about giving yourself an injection? | Feelings about giving self-injections |  |
| 4. How confident are you about giving yourself an injection in the right way? |  | Self-confidence |
| 5. How confident are you about giving yourself an injection in a clean and sterile way? |  |  |
| 6. How confident are you about giving yourself an injection safely? |  |  |
| 7. Does your current way of taking your medication make you feel in control of your disease? |  | **DELETED** |
| 8. Overall, how satisfied are you with your current way of taking your medication? | Satisfaction with self-injection | Satisfaction with self-injection |
