# Supplementary material for: Assessment of self-injection experience in patients with rheumatoid arthritis: psychometric validation of the Self-Injection Assessment Questionnaire (SIAQ)
Source: Health Qual Life Outcomes. 2011 Jan 13;9:2. doi: 10.1186/1477-7525-9-2 (PMC3027089; doi:10.1186/1477-7525-9-2)
Supplement: Additional file 2 — Self-Injection Assessment Questionnaire© (SIAQ) version 2.0. [file 1477-7525-9-2-S2.DOC]

# Additional file 2

# SELF-INJECTION ASSESSMENT QUESTIONNAIRE (SIAQ)

# - PRE-Self-Injection -

# Introduction

The following questions ask about injections in general and your feelings about giving yourself an injection.

Thank you for completing this questionnaire by yourself, preferably in a quiet environment. Take as much time as you need to complete it. There are no right or wrong answers. Your answers will remain strictly confidential and anonymous.

Please answer each question below by checking the box that best represents your opinion (Check only one box per question).

1. In general, how afraid are you of needles?

| Not at all | A little | Moderately | Very | Extremely |
| --- | --- | --- | --- | --- |

1. In general, how afraid are you of having an injection?

| Not at all | A little | Moderately | Very | Extremely |
| --- | --- | --- | --- | --- |

1. How anxious do you feel about giving **yourself** an injection?

| Not at all | A little | Moderately | Very | Extremely |
| --- | --- | --- | --- | --- |

1. How confident are you about giving yourself an injection in **the right way**?

| Not at all | A little | Moderately | Very | Extremely |
| --- | --- | --- | --- | --- |

1. How confident are you about giving yourself an injection in a **clean and sterile way**?

| Not at all | A little | Moderately | Very | Extremely |
| --- | --- | --- | --- | --- |

1. How confident are you about giving yourself an injection **safely**?

| Not at all | A little | Moderately | Very | Extremely |
| --- | --- | --- | --- | --- |

7. Overall, how satisfied are you with your current way of taking your medication?

| Very dissatisfied | Dissatisfied | Neither dissatisfied  nor satisfied | Satisfied | Very satisfied |
| --- | --- | --- | --- | --- |

**Thank you for completing this questionnaire**

**SELF-INJECTION ASSESSMENT QUESTIONNAIRE (SIAQ)**

**- POST-Self-Injection -**

**Introduction**

The following questions concern the self-injection of your medication and must be answered after giving yourself an injection.

Thank you for completing this questionnaire by yourself, preferably in a quiet environment. Take as much time as you need to complete it. There are no right or wrong answers. Your answers will remain strictly confidential and anonymous.

**Feelings about injections**

The following questions concern your **feelings about** **injections**.

Please answer each question below by checking the box that best represents your opinion (Check only one box per question).

In general, how afraid are you of needles?

| Not at all | A little | Moderately | Very | Extremely |
| --- | --- | --- | --- | --- |

In general, how afraid are you of having an injection?

| Not at all | A little | Moderately | Very | Extremely |
| --- | --- | --- | --- | --- |

How anxious do you feel about giving **yourself** an injection?

| Not at all | A little | Moderately | Very | Extremely |
| --- | --- | --- | --- | --- |

**Self-image**

The following question concerns your **self-image**.

Please answer the question below by checking the box that best represents your opinion (Check only one box).

How embarrassed would you feel if someone saw you with the self-injection device?

| Not at all | A little | Moderately | Very | Extremely |
| --- | --- | --- | --- | --- |

**Self-confidence**

The following questions concern your **confidence** about giving yourself an injection.

Please answer each question below by checking the box that best represents your opinion (Check only one box per question).

How confident are you about giving yourself an injection in **the right way**?

| Not at all | A little | Moderately | Very | Extremely |
| --- | --- | --- | --- | --- |

How confident are you about giving yourself an injection in a **clean and sterile way**?

| Not at all | A little | Moderately | Very | Extremely |
| --- | --- | --- | --- | --- |

How confident are you about giving yourself an injection **safely**?

| Not at all | A little | Moderately | Very | Extremely |
| --- | --- | --- | --- | --- |

**Pain and skin reactions during or after the injection**

The following questions ask about **pain and skin reactions** you may have experienced during or after the injection.

Please answer each question below by checking the box that best represents your opinion (Check only one box per question).

| During and/or after the injection, how bothered were you by: | Not at all | A little | Moderately | Very | Extremely |
| --- | --- | --- | --- | --- | --- |
| 1. **pain**? |  |  |  |  |  |
| 1. **burning sensation**? |  |  |  |  |  |
| 1. **cold sensation**? |  |  |  |  |  |

| During and/or after the injection, how bothered were you by: | Not at all | A little | Moderately | Very | Extremely |
| --- | --- | --- | --- | --- | --- |
| - 1. **itching** at the  injection site? |  |  |  |  |  |
| - 1. **redness** at the injection site? |  |  |  |  |  |
| - 1. **swelling** at the injection site? |  |  |  |  |  |
| - 1. **bruising** at the injection site? |  |  |  |  |  |
| - 1. **hardening** at the injection site? |  |  |  |  |  |

**Ease of Use of the self-injection device**

The following questions ask about the **ease of use** of the self-injection device.

Please answer each question below by checking the box that best represents your opinion (Check only one box per question).

| How difficult or easy was it to: | Very difficult | Difficult | Somewhat difficult | Somewhat easy | Easy | Very easy |
| --- | --- | --- | --- | --- | --- | --- |
| remove the cap? |  |  |  |  |  |  |
| depress the plunger or button on the device? |  |  |  |  |  |  |
| administer the injection without any help? |  |  |  |  |  |  |
| use the self-injection device? |  |  |  |  |  |  |

How does the device fit in your hand?

| Very uncomfortably | Uncomfortably | Somewhat uncomfortably | Somewhat comfortably | Comfortably | Very comfortably |
| --- | --- | --- | --- | --- | --- |

**Satisfaction with self-injection**

The following questions ask about your **satisfaction** with self-injection.

Please answer each question below by checking the box that best represents your opinion (Check only one box per question).

How easy was it to give yourself an injection?

| Not at all | A little | Moderately | Very | Extremely |
| --- | --- | --- | --- | --- |

How satisfied are you with **how often** you give yourself an injection?

| Very dissatisfied | Dissatisfied | Neither dissatisfied  nor satisfied | Satisfied | Very satisfied |
| --- | --- | --- | --- | --- |

How satisfied are you with the **time it takes to inject** the medication?

| Very dissatisfied | Dissatisfied | Neither dissatisfied  nor satisfied | Satisfied | Very satisfied |
| --- | --- | --- | --- | --- |

Overall, how satisfied are you with your current way of taking your medication (self‑injection)?

| Very dissatisfied | Dissatisfied | Neither dissatisfied  nor satisfied | Satisfied | Very satisfied |
| --- | --- | --- | --- | --- |

Overall, how convenient is the self-injection device?

| Very  inconvenient | Inconvenient | Neither inconvenient  nor convenient | Convenient | Very convenient |
| --- | --- | --- | --- | --- |

After this study, would you choose to continue self-injecting your medication?

| Definitely not | Probably not | I don’t know | Yes, probably | Yes, definitely |
| --- | --- | --- | --- | --- |

After this study, how confident would you be to give yourself injections at home?

| Not at all | A little | Moderately | Very | Extremely |
| --- | --- | --- | --- | --- |

**Thank you for completing this questionnaire**
